# Emergence of Fosfomycin Resistance by Plasmid-Mediated *fos* Genes in Uropathogenic ESBL-Producing *E. coli* Isolates in Mexico

**DOI:** 10.3390/antibiotics11101383

**Published:** 2022-10-09

**Authors:** Mario Galindo-Méndez, Humberto Navarrete-Salazar, Francisco Baltazar-Jiménez, Eduardo Muñoz-de la Paz, María Fernanda Sánchez-Mawcinitt, Alexis Gómez-Pardo, Elvira Garza-González, Luis Alfredo Ponce-de-León-Garduño, Rafael Franco-Cendejas, Rayo Morfín-Otero, Fabián Rojas-Larios, Juan Pablo Mena-Ramírez, Cecilia Teresita Morales-de-la-Peña, Lourdes García-Mendoza, Elena Victoria Choy-Chang, Laura Karina Avilés-Benítez, Eduardo López-Gutiérrez, Jorge Luis Canizales-Oviedo, Nicolás Eric Barlandas-Rendón, Joyarib Yanelli Maldonado-Anicacio, Alina Aracely Rosales-García, Heidy Leticia Ostos-Cantú

**Affiliations:** 1 Laboratorios Galindo SC, Department of Microbiology, Oaxaca 68000, Mexico; 2 School of Medicine, Universidad Anáhuac Oaxaca, San Raymundo Jalpan 71248, Mexico; 3 Hospital General Balbuena, Ciudad de México 15970, Mexico; 4 Hospital Universitario Dr. José E. González, Universidad Autónoma de Nuevo León, Monterrey 64460, Mexico; 5 Instituto Nacional de Ciencias Médicas y Nutrición Salvador Zubirán, Ciudad de México 14080, Mexico; 6 Instituto Nacional de Rehabilitación Luis Guillermo Ibarra, Ciudad de México 14389, Mexico; 7 Hospital Civil de Guadalajara e Instituto de Patología Infecciosa, Guadalajara 44280, Mexico; 8 Hospital Regional Universitario de Colima, Colima 28085, Mexico; 9 Hospital General de Zona 21 Tepatitlán De Morelos, Centro Universitario de los Altos (CUALTOS), Universidad de Guadalajara, Tepatitlán de Morelos 47620, Mexico; 10 Hospital General con Especialidades Juan María de Salvatierra, La Paz 23085, Mexico; 11 Hospital Angeles Valle Oriente, San Pedro Garza García 66260, Mexico; 12 Hospital General de Zona No. 1, Tapachula 30700, Mexico; 13 Hospital Infantil de Morelia, Morelia 58000, Mexico; 14 Hospital Regional de Alta Especialidad de Oaxaca, San Bartolo Coyotepec 71256, Mexico; 15 Laboratorio Pueblo Nuevo, Centro Universitario de Salud, Universidad Autónoma de Nuevo León, Monterrey 66455, Mexico; 16 Laboratorio Bioclin, Chilpancingo 39000, Mexico; 17 Hospital general de Chilpancingo, Chilpancingo 39019, Mexico; 18 Hospital de Especialidades pediátrico de León, León 39900, Mexico; 19 Bioclinsa, Hospital Ginequito, Monterrey 64060, Mexico

**Keywords:** ESBL, antimicrobial resistance, fosfomycin resistance, CTX-M, ST131

## Abstract

Fosfomycin is currently a viable option against urinary tract infections, particularly against extended-spectrum β-lactamases (ESBL)-producing *E. coli*, due to its unique mechanism of action and its low resistance among bacteria. The objective of this study was to investigate two of the three most common mechanisms of resistance against this antibiotic among 350 ESBL-producing *E. coli* strains isolated from the urine of Mexican patients. The prevalence of fosfomycin resistance in our study was 10.9% (38/350). Of all resistant isolates analyzed, 23 (60.5%) were identified as *fos*-producing organisms, with 14 strains carrying *fosA3* and 9, *fosA1*. Additionally, 11 (28.9%) fosfomycin-resistant isolates presented resistance due to impaired antibiotic transport and 8 (21.0%) both mechanisms. No resistance mechanism investigated in the study was found on 12 strains. All 38 confirmed ESBL-producing isolates carried a *bla_CTX-M_* subtype, 36 (94.5%) belonged to the O25b-ST131 clone, and all of them were able to transfer the fosfomycin resistance trait to recipient strains horizontally. This is the first study in Mexico demonstrating a plasmid-mediated fosfomycin resistance mechanism among clinical *E. coli* strains. Since our results suggest a strong association among *fos* and *bla_CTX-M_* genes and ST131 clones in uropathogenic *E. coli*, plasmid-mediated fosfomycin resistance should be closely monitored.

## 1. Introduction

Urinary tract infections (UTIs) are one of the leading bacterial infections worldwide, infecting over 150 million people of both genders each year [1]. In Mexico, the number of reported cases of UTIs exceeds four million annually and represents the third cause of morbidity in the country [2].

*Enterobacterales*, specifically *Escherichia coli*, are the most common cause of UTIs. In recent decades, treatment against these infections has become more complicated, mainly because of the increasing prevalence of antimicrobial resistance among uropathogenic *E. coli* strains. This increase in resistance is particularly due to the production of extended-spectrum β-lactamases (ESBL), mainly due to the CTX-M type, and to the global spread of the recently emerged, multidrug-resistant ST131 clone, predominantly serogroup O25b. This clone is the currently dominant extraintestinal pathogenic *E. coli* and the most common multidrug-resistant (MDR) high-risk clone associated with UTIs around the world [3,4]. In particular, this clone presents high levels of resistance to ciprofloxacin and aminoglycosides. Additionally, ST131 is commonly resistant to aztreonam, cephalosporins, and penicillins, brought upon the expression of the *bla_CTX-M_* gene, leading the World Health Organization (WHO) to consider it a critical priority pathogen [5].

Because of the growing concern about multidrug resistance among uropathogenic *E. coli* and the scarcity of new antimicrobial agents, the use of old antibiotics, such as fosfomycin, has reemerged as therapeutic options against this organism. Fosfomycin was developed over 40 years ago and inhibited the formation of the peptidoglycan precursor UDP N-acetylmuramic acid by forming a covalent bond with the Cys-115 residue in the active site [6]. Due to its mechanism of action, unrelated to that of other antibiotics, this unique antimicrobial is not expected to display cross-resistance with other antimicrobial agents. Reports of fosfomycin resistance among uropathogenic *E. coli* strains have varied depending on the epidemiology region of the study, with a generally low prevalence globally, indicating that fosfomycin is a feasible option in the treatment of UTIs [7,8]. Nevertheless, increasing resistance rates against this drug have been recently reported in some areas of the world, particularly in ESBL-producing *E. coli* [9,10].

Three different resistance mechanisms against fosfomycin have been demonstrated: (a) overexpression or mutations of MurA, the antibiotic target enzyme; (b) deficiencies in the expression of membrane transporter proteins (GlpT or UhpT), and (c) the expression of fosfomycin-inactivating enzymes by the addition of glutathione to the epoxide ring of the molecule [11]. The latter mechanism is mediated in *Enterobacterales* by different *fosA* and *fosC2* genes that have been reported in transposon elements and conjugative plasmids, accelerating the dissemination of fosfomycin resistance among bacteria. In *E. coli*, the most prevalent of the *fos* genes is *fosA3*, which is mostly associated with *bla_CTX-M_* and has been broadly reported in East Asia [10] but has spread to different countries in Europe [7,8,12], South America [13], and the United States [14].

Different studies in Mexico have assessed the fosfomycin resistance rates among uropathogenic *E. coli* strains [15,16]; however, none reported the molecular mechanism of resistance against this agent. Furthermore, information on the incidence of plasmid-mediated fosfomycin resistance in ESBL-producing *E. coli* strains isolated from urine is lacking in our country. In this study, we aimed to assess the in vitro activity of fosfomycin against uropathogenic *E. coli* isolated in Mexico and to study the presence of two of the most common mechanisms of resistance against this agent: antibiotic transport deficiencies and plasmid-mediated fosfomycin resistance.

## 2. Results

### 2.1. Fosfomycin Resistance, bla_CTX-M_, and ST131-O25b Prevalence

A total of 350 uropathogenic ESBL-producing *E. coli* strains were included. Fosfomycin resistance was confirmed in 38 (10.9%) strains. The MIC of fosfomycin resistant strains ranged from 128 to 2048 mg/L with 27 isolates (71.1%) showing an MIC ≥ 512 mg/L and 21 (55.3%) with an MIC ≥ 1024 mg/L (Table 1). All 38 fosfomycin-resistant strains carried *a bla_CTX-M_* gene, and 36 (94.7%) of them belonged to the O25b-ST131 clone.

### 2.2. Fosfomycin Resistance Mechanisms

The mechanisms of resistance of the 38 fosfomycin-resistant *E. coli* strains are summarized in Table 2; no mechanism was identified for 12 of them. Of all the resistant isolates analyzed, 23 (60.5%) were identified as *fos*-producing organisms, with 14 strains carrying *fosA3* and 9 carrying *fosA1*; no strains were found to carry *fosA4*, *fosA5*, *fosA6,* or *fosC2*. Additionally, 11 (28.9%) fosfomycin-resistant isolates presented resistance due to impaired antibiotic transport and 8 (21.0%) presented both mechanisms. All 11 strains harboring a fosfomycin transport deficiency were auxotrophic only to G3P. No correlation between the type of fosfomycin resistance and the MIC was found (Table 1).

### 2.3. Conjugation Experiments

Conjugation experiments were carried out on all *fosA3* and *fosA1*-positive strains, with 100% transmission of fosfomycin resistance to the recipient J53 *E. coli* strain. All transconjugants were shown to be ESBL producers by the CLSI confirmatory test [17], PCR positive for the respective *fos* and *bla_CTX-M_* genes of the donor bacterial cell, and de novo resistant to fosfomycin, as assessed by the agar dilution method (>64 ug/mL). All transconjugants presented either identical or a two-fold lower MIC than that of the parental strain.

## 3. Discussion

### 3.1. Fosfomycin Resistance Prevalence

The worldwide prevalence of urinary tract infections caused by ESBL-producing *E. coli* has been on the rise, thus complicating treatment against such infections. Despite the high mutation rates under experimental conditions of this organism that can lead to resistance against fosfomycin, resistance rates among uropathogenic *E. coli* against this agent are low [7,8,14,15,18], thus allowing this “forgotten” antibiotic to make a comeback as an empirical therapy against UTIs. 

In the current study, a total of 350 ESBL-producing *E. coli* uropathogenic strains isolated from patients from 18 centers in 13 different cities in Mexico were included and the rate of resistance found among them against fosfomycin was 10.9% (38/350). This figure is higher than those found in other countries [7,8,12,14,19] but similar to those reported in 2018 in a private hospital in Morelia, Mexico [20] and in a small study performed the same year in the city of Oaxaca, Mexico [15]. The fosfomycin resistant rates reported in the current study among uropathogenic *E. coli* and those found in the latter studies are all lower than those reported against oral antibiotics commonly used to treat UTIs in Mexico, such as ciprofloxacin and cephalosporins [15,20,21]. The results of the current study are in agreement with the fosfomycin resistance rates found in Europe in the “Surveillance of sUsceptibility and Resistance to Fosfomycin in comparison with other antimicrobial agents study” (SURF) [22], where fosfomycin and nitrofurantoin were the oral antibiotics with the lowest resistance against uropathogenic *E. coli*. However, larger studies in Mexico are needed to confirm if fosfomycin is a viable option in the empiric treatment of UTIs, as suggested by our in vitro results. 

### 3.2. Fosfomycin Resistance Mechanisms

In the past, several studies have been published in Mexico showing the resistance rates of uropathogenic *E. coli* against fosfomycin [15,20,21], but the mechanisms of resistance against this agent were not described. In the current study, two of the three known mechanisms of fosfomycin resistance were investigated: antibiotic transport deficiencies and *fos*-mediated plasmid resistance. The results of this study showed that one of these two mechanisms, alone or together, was present in 26 of the 38 ESBL-producing *E. coli* strains resistant to this antibiotic. In the 12 strains that did not present transport deficiencies and no *fos* genes were identified, the most likely mechanism of their resistance to fosfomycin was the modification of the target protein of the antibiotic, MurA, or the presence of a *fos* gen different from those included in the present study; thus, future studies must include whether mutations in MurA contribute to fosfomycin resistance in Mexico. However, unknown mechanisms of fosfomycin resistance cannot be discarded as the cause of resistance among these 12 clones and require further evaluation.

### 3.3. Fosfomycin Transport Deficiencies

The antibiotic fosfomycin enters the bacterial cell by active transport via two proteins, GlpT or UhpT; the former protein is constitutively expressed, whereas the latter is induced by extracellular G6P. Of the 38 *E. coli* strains resistant to fosfomycin, 11 were unable to grow in minimal media provided with a sole source of carbon, either G3P or G6P. As 100% of these strains showed no growth on G3P media, these results suggest that fosfomycin resistance is due to a functionless glpT transporter, mutations in any of its regulators (*uhpA*, *cyaA*, and *ptsI*), or because of a combination of mutations on the genes encoding the GlpT system, as shown by Cattoir et al. [23].

### 3.4. Plasmid-Mediated Fosfomycin Resistance

In the early years of research on fosfomycin resistance in *E. coli*, the predominant mechanism was that of chromosomal mutations in the antibiotic transporter systems [24,25]; however, the molecular mechanisms of resistance against this agent have shifted toward a higher prevalence of plasmid-mediated resistance [7,8,12,14,19,26,27]. In the current study, in addition to the investigation of transport deficiency as the mechanism of fosfomycin resistance among ESBL-producing uropathogenic *E. coli*, the presence of plasmid-mediated genes, *fos* genes, was also investigated. Our results indicate that 23/38 (60.5%) strains carried one of the *fos* genes included in the investigation, making *fos*-mediated resistance the major fosfomycin resistance mechanism found in this study. However, larger studies are needed to corroborate whether plasmid-mediated resistance is indeed the most prevalent fosfomycin resistance mechanism in Mexico. The results of this investigation are in agreement with others, indicating that fosfomycin resistance due to *fosA* genes is spreading around the globe [7,8,12,14,19,26,27]. The prevalence of *fosA* genes in our isolates of fosfomycin-resistant ESBL-producing *E. coli* was high (60.5%), similar to previous studies performed in Japan [27] and China [19], but contrary to those found in Europe [7,8]. These results indicate that the prevalence of *fos*-mediated resistance depends on the geographical area of the study.

The first plasmid-mediated *fosA* genes, *fosA1*, were described in the late 1980s [28]. To date, there are eight different *fosA* genes that have been described in *E. coli* [26], with the *fosA3* gene being the most prevalent in the family. *fosA3* is believed to have originated in Asia and was likely mobilized from the chromosome of *K. georgiana* [29]. Of the 38 strains that were identified as fosfomycin resistant in our study, 23 carried a *fosA* gene, with 14 carrying *fosA3* and 9 carrying *fosA1*. Based on these data, these results indicate that fosfomycin resistance in ESBL-producing isolates of uropathogenic *E. coli* caused by *fos* genes has spread throughout Mexico and, consistent with reports from different studies around the world [13,14,19,27], we found that in our setting *fosA3* is the most prevalent plasmid-mediated mechanism of fosfomycin resistance.

In the current study, to our knowledge, the presence of *fosA* genes is reported for the first time in clinical isolates in Mexico. As insertion sequences are often found on plasmids carrying these genes, mobilization of such antibiotic resistance traits among different bacteria can occur, allowing its dissemination. In this investigation, 100% of the fosfomycin-resistant ESBL-carrying strains were able to horizontally transmit their resistance to the recipient *E. coli* strain, suggesting the possibility that conjugation might aid in the transmission of fosfomycin resistance among bacteria. Further studies are needed to determine the frequency of the horizontal transmission of this resistant trait in nature, specifically in the gut of animals and humans, as it has been shown that certain conditions such as oxygen, bile salts, and NaCl concentrations in the intestine favor transfer rates of certain plasmids but inhibit transmission of others [30].

### 3.5. CTX-M Prevalence

The results in this study demonstrate that 100% of the fosfomycin-resistant ESBL-producing *E. coli* strains included in this study were CTX-M producers, in agreement with the results reported in China [10], also performed on strains isolated from urine, where a high percentage of similar organisms carried the *bla_CTX-M_* gene, suggesting a likely association between the two resistance traits. Different studies in Asia [31,32,33] have demonstrated that *fosA* and *bla_CTX-M_* genes can be coharbored on conjugative plasmids in *E. coli*, thus allowing the possible simultaneous dissemination of these antibiotic resistance determinants among bacteria [29].

The association of *bla_CTX-M_* and *fos* genes coharbored in the same organism is worrisome. In the last few years, the world has witnessed a major increase in the prevalence of community-acquired urinary tract infections caused by CTX-M-producing *E. coli*. This spreading around the world, known as the “CTX-M pandemic,” along with an already high prescription of cephalosporins and an increase in the use of fosfomycin, can provide an opportunity to select for *fosA* producers, leading to possible future treatment failures with this antimicrobial. Furthermore, Tseng et al. [34] and Jiang et al. [35] demonstrated in China that the same transposable elements surrounding *fosA*- and ESBL-carrying plasmids in humans could be found in pig and chicken isolates, suggesting that these genes could be transferred from domestic animals to humans, increasing the risk of human colonization with *fos*-carrying *E. coli*. The horizontal transmission of these plasmid-mediated resistance genes is of high risk for their spread, and since the biological cost of plasmid-mediated fosfomycin resistance is lower than that induced by mutations in the chromosome [36], the global spread of this resistance mechanism should be closely monitored.

### 3.6. ST131-O25b Clone

Currently, *E. coli* ST131 is recognized as the major lineage responsible for the spread of *bla_CTX-M_* -ESBL genes. Additionally, most ST131 isolates are resistant to fluoroquinolones, and many are co-resistant to trimethoprim-sulfamethoxazole and aminoglycosides. The results of our investigation show that a very high percentage of *fos*-carrying *E. coli* strains included in this study, 36/38 (94.7%), belonged to the O25b-ST131 clone. These results suggest that in addition to being a carrier of previously reported resistant traits, this clone can also acquire plasmid-mediated fosfomycin resistance genes. As previously indicated by Oteo et al. in Spain, a CTX-M-producing E. coli O25b-ST131 strain can acquire fosfomycin resistance genes, spread in the community, and increase the prevalence of resistance against this agent among uropathogenic *E. coli* [9].

As ST131 has been shown to be transmitted due to person-to-person contact and through consumption of food [4], lack of hygiene plays a role in the transmission of this multiresistant clone, which suggests that less privileged areas of the world might see an increase in the prevalence of this clone. If *fosA*-carrying O25b-ST131 *E. coli* clones expand to different geographical areas, other antibiotics, such as ciprofloxacin, which is largely used to treat UTIs in different countries empirically, can co-select for fosfomycin-resistant *E. coli* strains and, thus, contribute to the spread of resistance against this agent.

Limitations of the current study included that, although strains from different regions of the country were incorporated, the sample size was somewhat small, and our results do not necessarily apply to the entire country. A second limitation was that the chromosomal mechanisms of fosfomycin resistance due to *MurA* mutations were not included in the study; thus, its contribution to the overall understanding of the mechanism of resistance against this agent in Mexico is not known. Finally, the genetic relatedness of the strains included in the study and the presence of *bla_CTX-M_* in these clones were not investigated.

### 3.7. Conclusions

In conclusion, to our knowledge, this is the first report of the presence of plasmid-mediated fosfomycin resistance genes (*fosA*) in *E. coli* isolated from clinical specimens of humans in Mexico. The results of this study suggest that this mechanism may be an important driver of resistance against fosfomycin in our country. Additionally, our results demonstrate that fosfomycin resistance in our setting is associated with *bla_CTX-M_* genes and ST131 clones and can be horizontally transmitted to other bacteria, situations that alert the medical community for possible future dissemination of fosfomycin resistance, which should be routinely surveilled.

## 4. Materials and Methods

### 4.1. Bacterial Strains

A total of 350 non-duplicate ESBL-producing *E. coli* strains isolated from urine in counts of ≥100,000 UFC/mL were kindly provided by 18 different laboratories distributed across Mexico from the Red Tematica de Investigacion y Vigilancia Epidemiológica (INVIFAR network) and were included in this study. All strains had been previously isolated from adult patients (≥18 years) who submitted a urine sample for culture, following the physician´s instructions, due to complaints of symptoms of urinary tract infections. Identification of all strains was confirmed using API 20E strips (bioMérieux, Marcy-l’Étoile, France); ESBL production was confirmed using the Clinical Laboratory Standard Institute (CLSI) phenotypic confirmatory test (17).

### 4.2. Antimicrobial Susceptibility

The susceptibility of all *E. coli* isolates to fosfomycin was initially screened by the disk diffusion method according to CLSI guidelines, using 200 μg fosfomycin disks containing 50 μg of glucose-6-phosphate on Mueller Hinton agar [17]. The Minimum Inhibitory Concentration (MIC) of strains showing zone diameters above CLSI breakpoints (≥16 mm) were further determined in duplicate by the agar dilution method [17]. Fosfomycin disodium salt (Sigma Aldrich) was added to Mueller–Hinton agar containing 25 mg/L glucose-6-phosphate to obtain two-fold dilutions, spanning concentrations from 16 to 2048 mg/L.

### 4.3. Utilization of Carbohydrates

All *E. coli* strains with a MIC above the CLSI breakpoint (>64 mg/L) were plated on M9 minimal medium agar supplemented with either glyceraldehyde 3-phosphate (G3P) or glucose-6-phosphate (G6P) at 0.2% (w/v) as the sole source of carbon. Growth was determined after incubation at 35 ± 2 °C for 48 h; no growth on either plate indicated impaired transport of fosfomycin [37].

### 4.4. PCR Amplification

Plasmid DNA was extracted from fresh bacterial cells using QIAprep Spin Miniprep Kit (QIAGEN), following the manufacturer´s instructions. To detect *fos* genes, previously described primers for *fosA1*, *fosA3*, *fosA4*, *fosA5*, *fosA6*, and *fosC2* and PCR conditions were used (Table 3). In the strains that amplified the *fos* genes, sequencing was conducted.

Detection of the *E. coli* O25b-ST131 clones was performed using primers for the *pabB* gene [38]. To detect the presence of the *bla_CTX-M_* gene, primers and PCR conditions were used as previously described [39] (Table 3).

### 4.5. Conjugation Experiments

Conjugation experiments were carried out using *E. coli* J53, a sodium azide resistant strain, as the recipient organism, following the broth mating method. Transconjugants were selected on Mueller Hinton agar plates containing 100 mg/L sodium azide, 64 mg/L fosfomycin, and 25 mg/L G6P. The presence of *fos* genes in transconjugants was assessed by PCR and antimicrobial susceptibility testing utilizing the agar dilution method as suggested by CLSI [17]. The transmission of the *bla_CTX-M_* gene to transconjugants was confirmed by PCR [39] and its ESBL phenotype by the CLSI confirmatory method [17].

## Figures and Tables

**Table 1:** Minimum inhibitory concentrations (MIC) of uropathogenic ESBL-producing *E. coli* strains and their mechanisms of fosfomycin resistance.

Strain	Mechanism of Resistance	MIC (ug/mL) Parental Strain
24 1249 1268 1292 1430 308 1048 1256 1327 1463 326 1360 1097 1316 335	*fos* mediated resistance alone	2048 2048 2048 2048 2048 1024 1024 1024 1024 1024 512 512 256 256 128
1075 283 964	Impaired fosfomycin transport alone	2048 128 128
149 1295 1309 1432 118 85 871 733	Impaired fosfomycin transport + *fos*-mediated resistance	2048 2048 2048 2048 512 256 128 256
141 455 532 866 1163 418 1312 143 201 157 1315 248	No mechanism identified	2048 2048 2048 2048 2048 1024 1024 512 512 256 256 128

**Table 2:** Mechanisms of fosfomycin resistance identified in uropathogenic ESBL-producing *E. coli* strains.

Mechanism of Resistance	Number of Isolates
*fos* genes *fos* genes alone	23/38 15/38
Impaired fosfomycin transport Impaired transport alone Impaired fosfomycin transport + *fos* genes No mechanism identified	11/38 3/38 8/38 12/38

**Table 3:** Primers and PCR conditions used to determine the presence of *fos* and *bla_CTX-M_* genes and the O25b-ST131 clone.

Amplified Gene	Primers	Reference
*fosA1*	F 5′-ATC TGT GGG TCT GCC TGT CGT-3′ R 5′-ATG CCC GCA TAG GGC TTC T-3′	[12]
*fosA3*	F 5′-CCTGGCATTTTATCAGCAGT-3′ R 5′-CGGTTATCTTTCCATACCTCAG-3′	[12]
*fosA4*	F 5′-CTG GCG TTT TAT CAG CGGTT-3′ R 5′-CTTCGCTGCGGTTGTCTTT-3′	[12]
*fosA5*	F 5′-TATTAGCGAAGCCGATTTTGC T-3′ R 5′-CCC CTT ATA CGG CTG CTC G-3′	[12]
*fosA6*	F 5′-CGAGCGTGGCGTTTTATCAG-3′ R 5′-GGCGAAGCTAGCAAAATCGG-3′	[12]
*fosC2*	F 5′-TGG AGG CTA CTT GGA TTT G-3′ R 5′-AGG CTA CCG CTA TGG ATT T-3′	[12]
*pabB*	F 5′-TCCAGCAGGTGCTGGATCGT-3′ R 5′-GCGAAATTTTTCGCCGTACTGT-3′	[38]
*bla_CTX-M_*	F 5’-TTTGCGATGTGCAGTACCAGTA-3′ R 5’-CGATATCGTTGGTGGTGCCATA-3′	[39]

## Data Availability

Not applicable.
